# Supplementary figures and images for: Lymphocytes influence Leishmania major pathogenesis in a strain-dependent manner
Source: PLoS Negl Trop Dis. 2019 Nov 18;13(11):e0007865. doi: 10.1371/journal.pntd.0007865 (PMC6886877; doi:10.1371/journal.pntd.0007865)

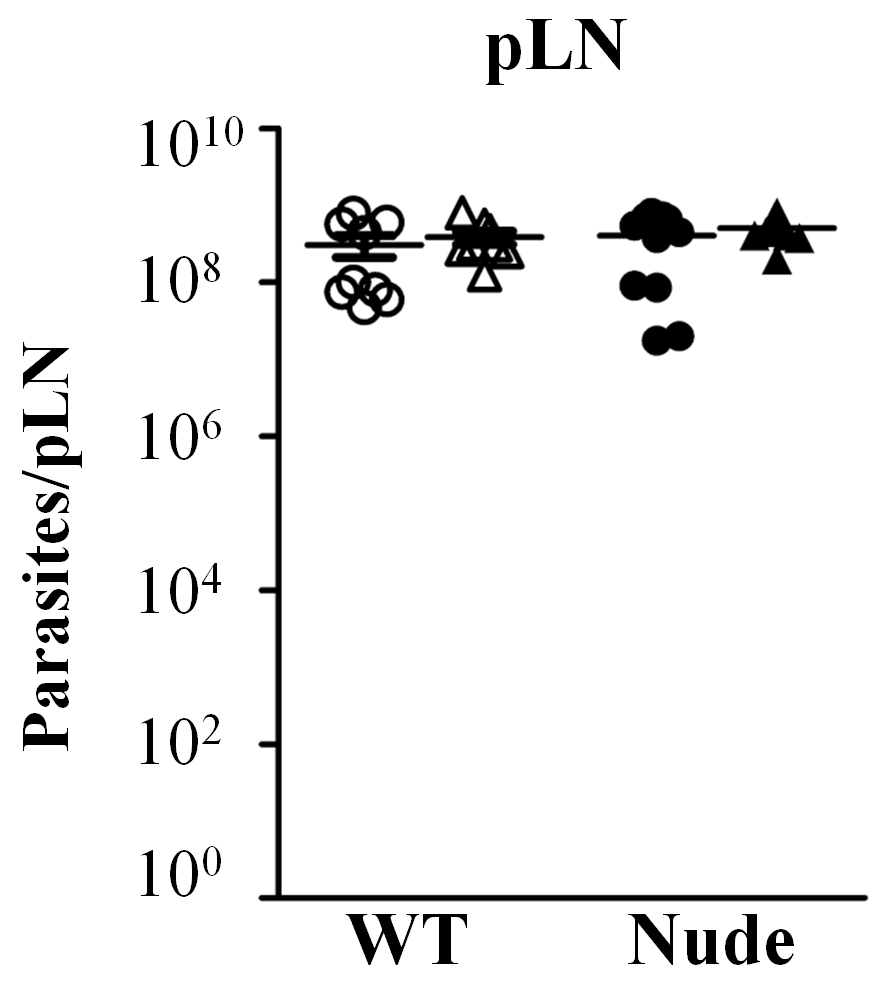

Supplement: S1 Fig — BALB/c WT and nude mice were infected with 5×10^6^ stationary phase promastigotes of L. major Friedlin or 5ASKH subcutaneously into the hind footpad. Parasite burden in popliteal lymph nodes (pLN) at 6 weeks post-infection. Each mouse footpad was homogenized in 10 ml culture media and cultured for 3 days then viable promastigotes were counted microscopically. Each group contains 8–11 mice. Symbols: BALB/c mice infected with L. major Friedlin ○ or 5ASKH △, BALB/c-nude mice infected with L. major Friedlin ● or 5ASKH ▲; Data are mean ± SEM. (TIF) [file pntd.0007865.s001.tif]

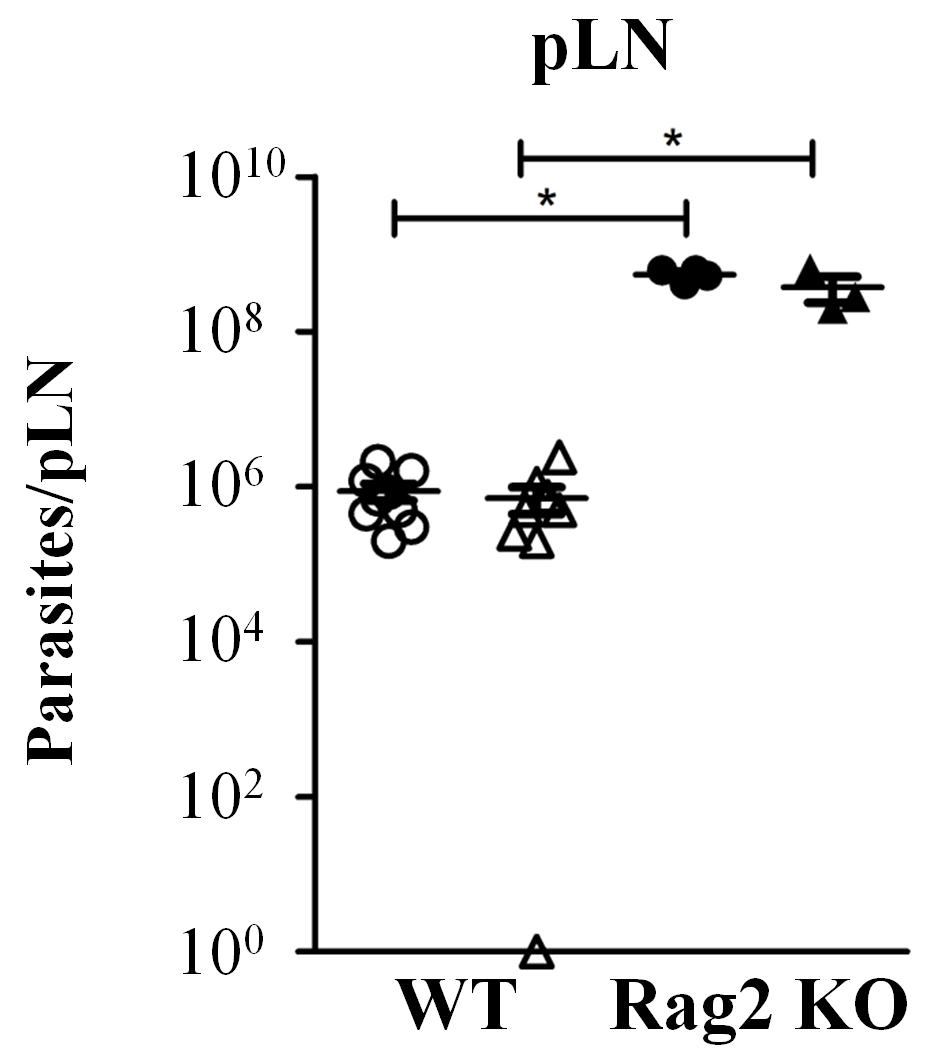

Supplement: S2 Fig — C57BL/6 WT and Rag2 KO mice were infected with 5×10^6^ stationary phase promastigotes of L. major Friedlin or 5ASKH subcutaneously into the hind footpad. Parasite burden in popliteal lymph nodes (pLN) at 6 weeks post-infection. Each mouse footpad was homogenized in 10 ml culture media and cultured for 3 days then viable promastigotes were counted microscopically. 3–9 mice per group. Symbols: wild-type mice infected with L. major Friedlin ○ or 5ASKH △, Rag2 KO mice infected with L. major Friedlin ● or 5ASKH ▲. Data are mean ± SEM. Results are representative of 2 independent experiments with a similar outcome. (TIF) [file pntd.0007865.s002.tif]

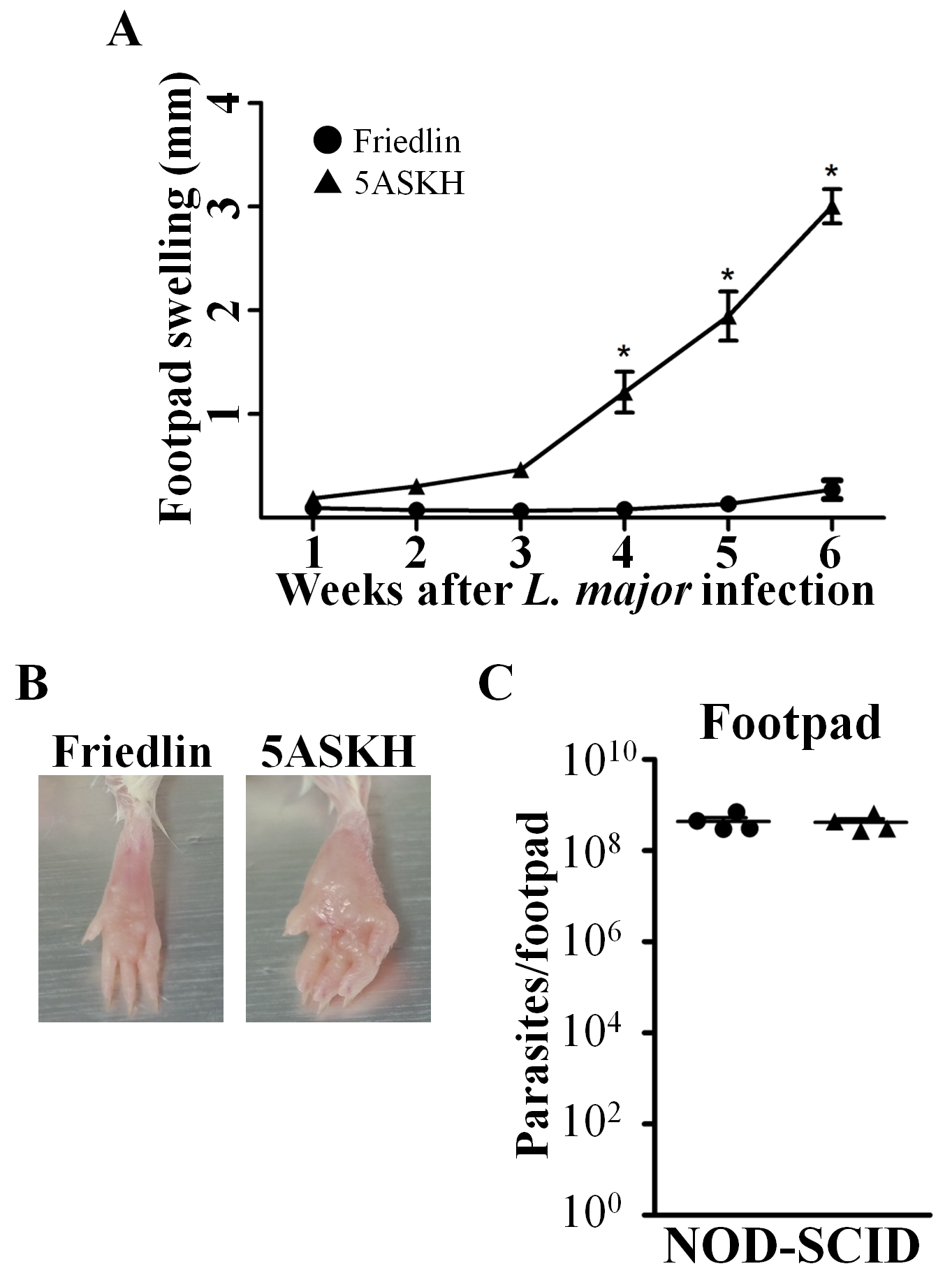

Supplement: S3 Fig — NOD-SCID mice were infected with 5×10^6^ stationary phase promastigotes of L. major Friedlin or 5ASKH subcutaneously into the hind footpad. (A) Footpad swelling after L. major infection. Symbols: NOD-SCID mice infected with L. major Friedlin ● or 5ASKH ▲; 8 mice per group. (B) Representative photographs of L. major infected footpad at 6 weeks post-infection. (C) Footpad parasite burden at 6 weeks post-infection. Each mouse footpad was homogenized in 10 ml culture media and cultured for 3 days then viable promastigotes were counted microscopically. 4–8 mice per group. Data are mean ± SEM. Results are representative of two independent experiments with a similar outcome. (TIF) [file pntd.0007865.s003.tif]

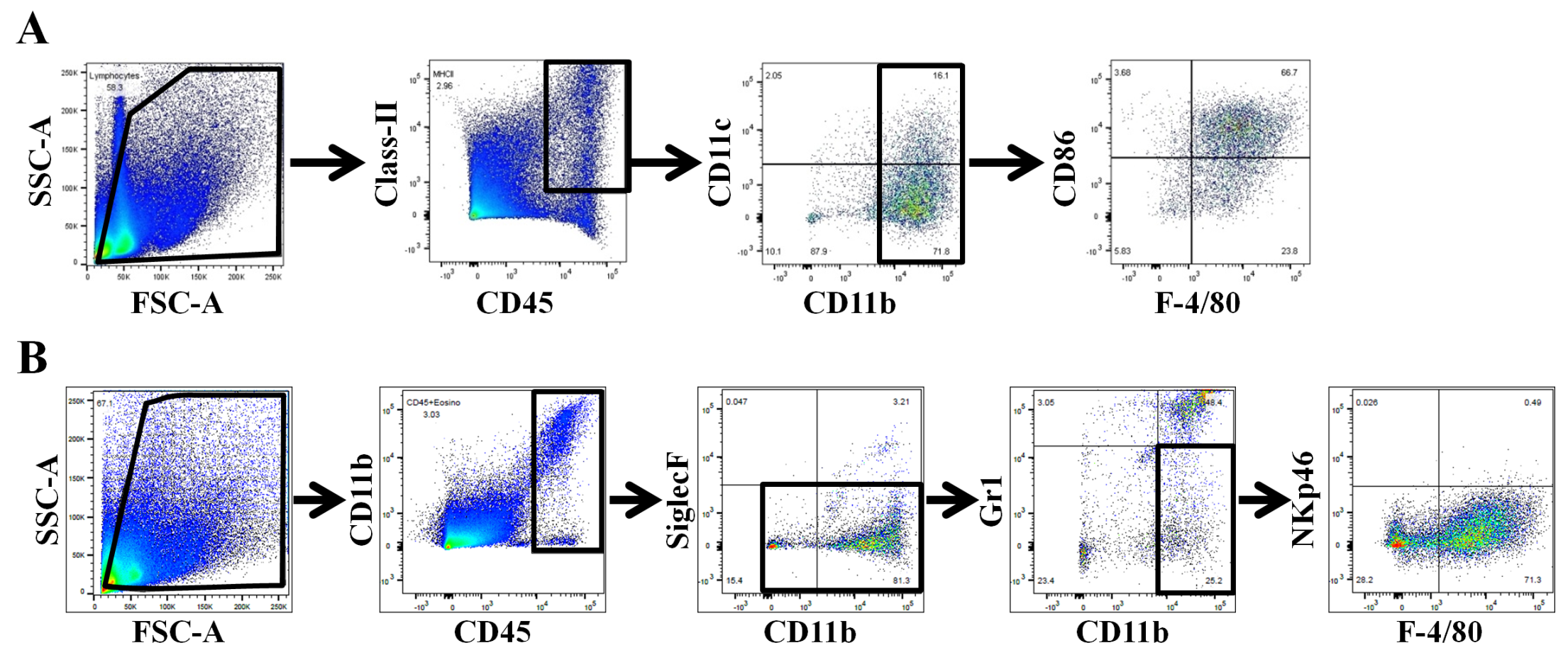

Supplement: S4 Fig — Rag2 KO C57BL/6 mice were infected with 5×10^6^ stationary phase promastigotes of L. major Friedlin or 5ASKH subcutaneously into the hind footpad. At 4 weeks post-infection, cells were isolated from mouse footpads and analyzed by flow cytometry. Gating strategies for (A) macrophages; (B) neutrophils, eosinophils, and NK cells. (TIF) [file pntd.0007865.s004.tif]

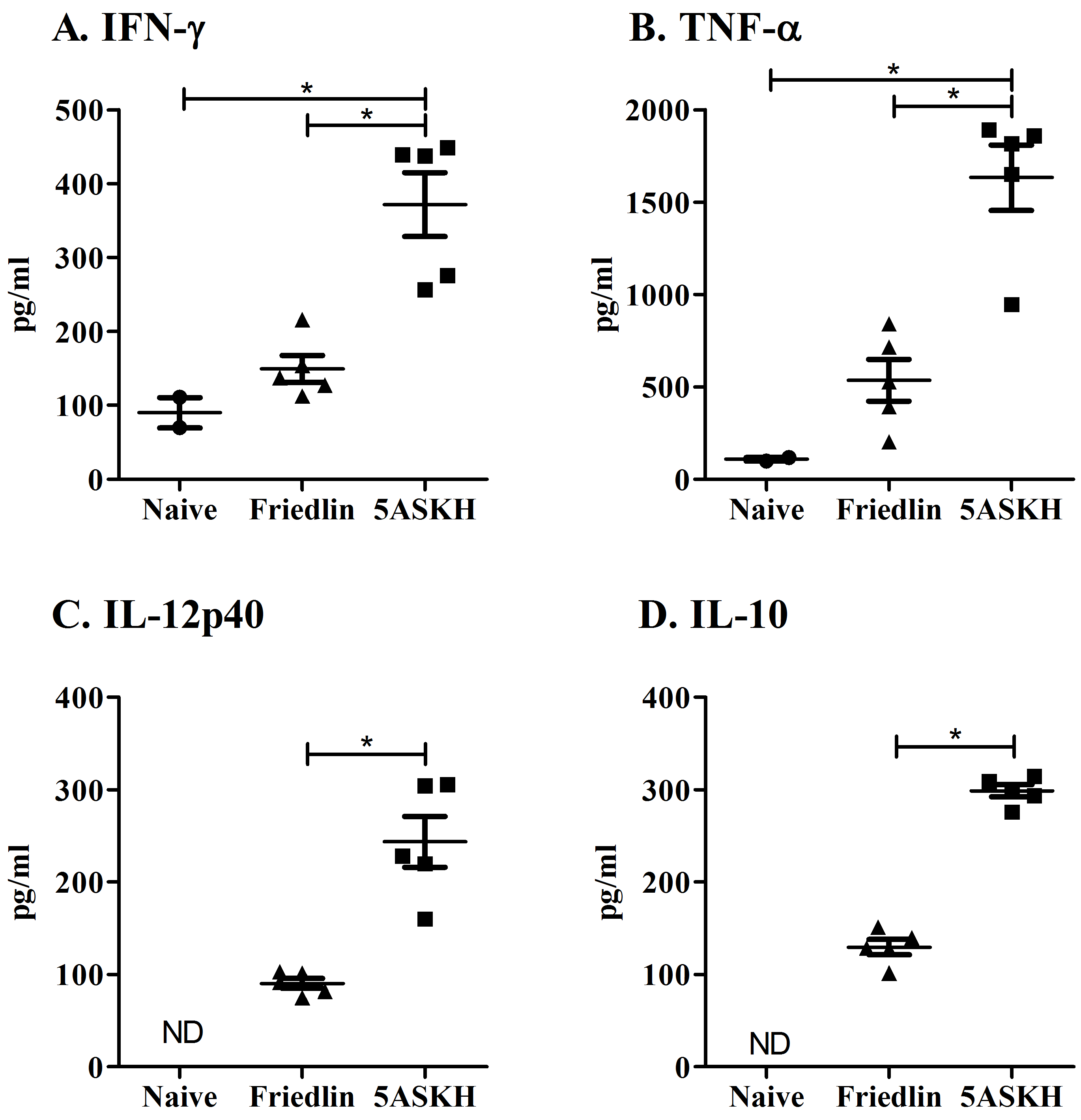

Supplement: S5 Fig — Rag2 KO C57BL/6 mice were infected with 5×10^6^ stationary phase promastigotes of L. major Friedlin or 5ASKH subcutaneously into the hind footpad. At 4 weeks post-infection cells were isolated from mouse footpads and stimulated with PMA-ionomycin for 3 days. Culture supernatants were analyzed for cytokines by ELISA. 3–4 mice per group. Data are mean ± SEM. *, P<0.05. (TIF) [file pntd.0007865.s005.tif]

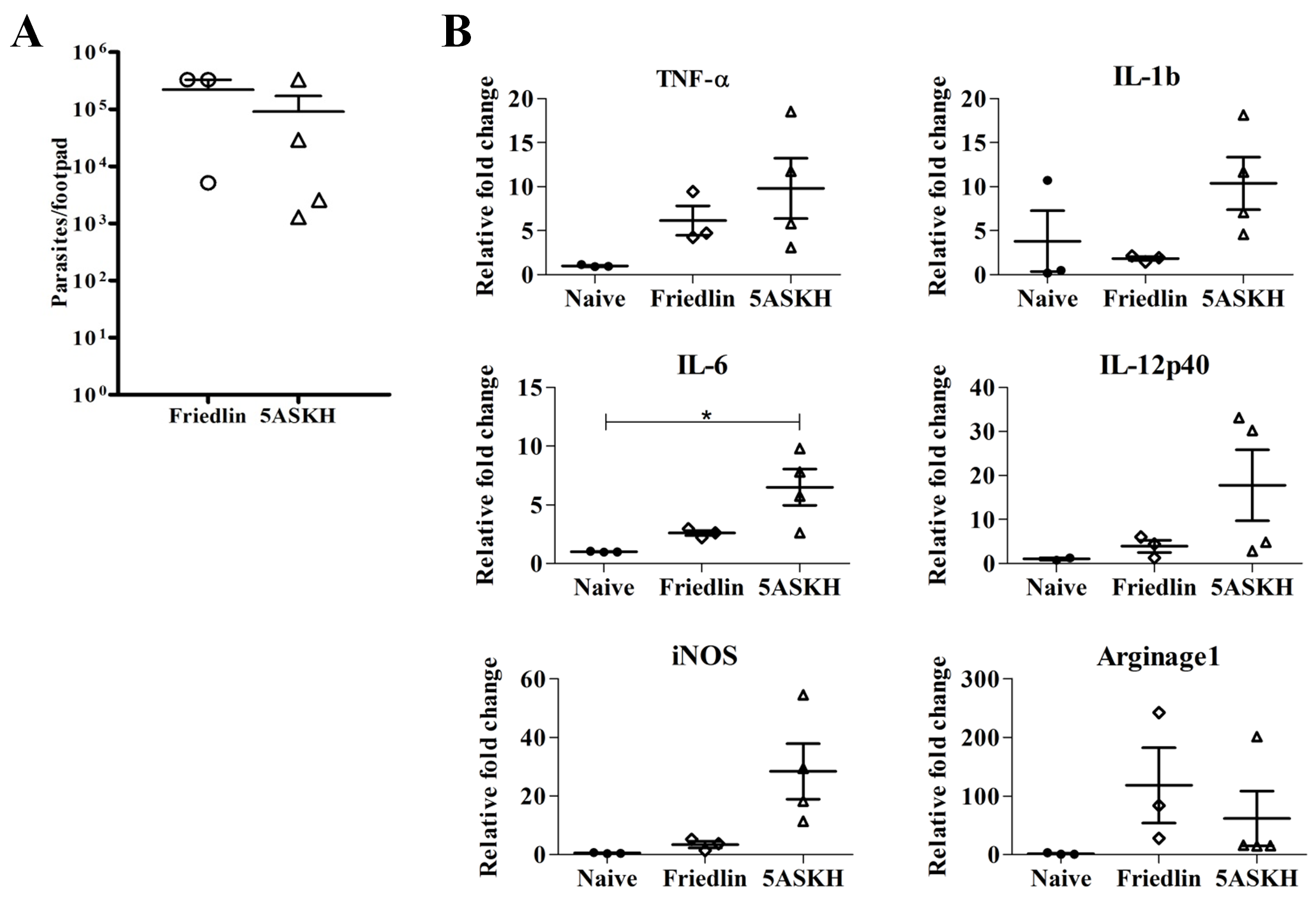

Supplement: S6 Fig — C57BL/6 mice were infected with 5×10^6^ stationary phase promastigotes of L. major Friedlin or 5ASKH subcutaneously into the hind footpad. At 4 weeks post-infection total RNA was isolated from mouse footpads and analyzed for mRNA of targeted molecules by real-time RT-PCR. (A) Footpad parasite burden at 4 weeks post-infection measured by limiting dilution assay. (B) mRNA expression in the infected footpad at 4 weeks post-infection. 3–4 mice per group. Data are mean ± SEM. (TIF) [file pntd.0007865.s006.tif]
