# Supplementary material for: Lymphocytes influence Leishmania major pathogenesis in a strain-dependent manner
Source: PLoS Negl Trop Dis. 2019 Nov 18;13(11):e0007865. doi: 10.1371/journal.pntd.0007865 (PMC6886877; doi:10.1371/journal.pntd.0007865)
Supplement: Attachment — Submitted filename: Response to reviewers.docx [file pntd.0007865.s007.docx]

**Responses to review comments:**

**Methods**

Reviewer #1: The studies by Abu Musa et al are of interest. Their findings help reconcile discrepant observations in the literature.

**Response**: Thank you for the inspiring comments.

Reviewer #2: The objectives of the study are clearly defined.

The study design is appropriate, but there are problems in the interpretation of data.

The same previous answer

The number of experiments and mice used in the experiments is fine.

The authors used ANOVA, but no test for normality of the data was done

No concerns about ethical or regulatory requirements

**Response**: Thank you for the comments. We hope we have improved our data interpretations, which is reflected in results (section 3) and discussion (section 4) sections. We had performed Shapiro-Wilk normality test to check normality of our data where applicable, which confirmed the data are mainly from normal distribution except a few experimental groups where the data are significantly deviated from normal distribution (now line number 211-212 in the track changes highlighted version) .

In Fig 1A, footpad swelling data of BALB/c mice at 1 and 3 weeks post-infection of *L. major* Friedlin have deviated from normal distribution. Footpad swelling data of BALB/c-nude mice at 2 weeks post-infection of *L. major* Friedlin have deviated from normal distribution.

In Fig 2A, footpad swelling data of C57BL/6 mice at 1-week post-infection of *L. major* Friedlin have deviated from normal distribution.

We have excluded those data sets deviated from normal distribution from ANOVA analysis (line 246-249; 282-284).

Reviewer #3: Although the methods are suitable, can the authors please clarify/rectify the following points?

1) In regards to Fig 1A, Fig 2A and S3A Fig, why was one-way ANOVA used to determine statistical significance?

Since their phenotype depends time and experimental condition, two-way ANOVA seems more appropriate.

**Response**: Thank you for the comment and suggestion. We performed two-way ANOVA analysis to determine statistical significance and changed Figs 1A, 2A and S3A. To fit our data with two-way ANOVA analysis, we removed a few data in Friedlin/5ASKH infected Rag2 KO mice, where we missed some mice to follow up until 6 weeks post-infection. The removal of a few Rag2 KO mice data was reflected in the graph (the groups of Rag2 KO mice infected with Friedlin/5ASKH in Fig 2A) (please see the updated figures 1A, 2A, S3A and line 213 in the track changes highlighted version).

2) In Figs 1A and 2A, errors bars are too thick, which makes it confusing for readers to properly visualize and assess the data. Since there are four different time series in the same graphs, the authors should use different colours to better differentiate the lines.

**Response**: Thank you for the comment and suggestion. We have reformatted Figs 1A and 2A with 4 different color lines for different time series of footpad swelling.

3) Numerical data in Figs 3-5 and S5 Fig should also be depicted in the form of scatter plots, as done in Fig 1C, S1 Fig and elsewhere.

**Response**: We have replaced bar graphs with scatter plots for Figs 3-5 and S5 Fig.

**Results**

Reviewer #1: Overall data and conclusions are clearly presented and discussed.

**Response**: Thank you for the comments.

Reviewer #2: Yes, it does.

The Figures presented low quality, it is not clear the statistical differences shown in the Figures

**Response**: We are uploading good quality figures into the online submission system of PLOS NTD. During conversion to PDF file figures quality getting down. We checked figure files using Preflight Analysis and Conversion Engine (PACE) digital diagnostic tool, which confirmed that figures meet PLOS requirements.

Reviewer #3:

1) Overall, results are laid out well, but the description of the data is often incorrect (see ‘Summary and General Comments’). For example, Figs 1-2 do not really show a ‘T lymphocyte-dependent pathogenesis by L. major Friedlin and independent in 5ASKH’, but rather whether lymphocytes worsen/help control disease by those strains.

**Response**: Thank you for the precious comment. According to your suggestion we modified our data interpretation. Our current statement is that lymphocytes influence (either exacerbate or ameliorate) the outcome of infection depending on *L. major* strains.

2) In Fig 1B, the photos pertaining to the 5ASKH strain do not recapitulate the data shown in 1A, which show that 5ASKH-induced lesions worsened in BALB/c nude mice. Since the photos in 1B show the opposite, perhaps the authors accidentally flipped them?

**Response**: Thank you for the comment. We have chosen a better representative photograph from 5ASKH infected wild-type BALB/c mice group (Fig 1B).

**Conclusions**

Reviewer #1: The paragraph beginning on line 361 discusses observations with the different Leishmania species; however, the authors may consider including a bit more information about the pedigree of both of the strains that were tested, in other to give some context of the diversity of disease presentations that may be seen in L. major endemic regions.

**Response**: Thank you for the suggestions. *L. major* Friedlin and 5ASKH were originally isolated from patients with CL in the old world, Israel and Turkmenistan respectively, then adapted to the conditions in the Laboratory and established as the strains (now line 436-439 in the track changes highlighted version).

Reviewer #2: The conclusions are supported by the data, but the discussion is a summary of the results. The limitations of analysis are not described.

Yes, they do, but some of the statements are not in agreement with the literature,

Yes, it is

**Response**: Thank you for the comments and suggestions. We believe we have improved our discussion (section 4) and conclusion (section 5) sections. We have included several limitations of our study in the discussion section as well (section 4) (now line 353, 356-358, 361-369, 373-376, 388-390, 393-407, 409-426, 436-439, 449-462, 465-469, 470, 476-478, 483-484).

Reviewer #3: The conclusions are partially supported by the authors’ findings. Section 5 (Conclusions) should focus on the positive and negative influence that lymphocytes have on the pathologies caused by the strains that the authors employed. As such, that section should be written concisely. The text pertaining to parasite virulence factors

and whatnot should be integrated within the main body of the Discussion (section 4).

**Response**: Thank you for comment and suggestion. According to your recommendation we have updated our conclusion section. Influence of lymphocytes in the pathogenesis caused by the strains has been included (now line 476-478, 483-484) in the conclusion section. In addition the text related to parasite virulence factors have been moved to the discussion section (now line 420-426).

**Editorial and Data Presentation Modifications**

Reviewer #1: No concerns noted

Reviewer #2: (No Response)

Reviewer #3: 1) Lines 379-392 ramble too much about the link that their mouse-based work has with Leishmania/HIV co-infected patients. In fact, lines 387-392 contain a very speculative and incoherent argument.

**Response**: We have removed the lines (449-462).

2) Authors should employ current and proper genetic terminology to describe their mouse strains throughout the manuscript.

**Response**: Thank you for comment and suggestion. We have included the mouse strains information in the “Materials and Methods” section (now line 123-127).

3) In the title, the word ‘the’ should be omitted. As per my major concerns (outlined in the ‘Summary and General Comments’ section), the word ‘requirement’ should be replaced by ‘influence’.

I suggest the following title: ‘Lymphocytes influence Leishmania major pathogenesis in a strain-dependent manner’.

**Response**: We sincerely appreciate your suggestion. We have understood the point well and changed the title as per your recommendation‘Lymphocytes influence *Leishmania major* pathogenesis in a strain-dependent manner’ (now line 2-7).

**Summary and General Comments**

Reviewer #1: The results of the study are significant as they bring to the fore observations that cutaneous disease presentation in endemic areas is variable. The variability could apparently be be due to strain differences within parasite species that impact immune responses.

**Response**: Thank you for comments.

Reviewer #2: The manuscript showed differences in the development of lesions by two different strains of L. major (Friedlin and 5ASKH) using different mouse strains such as BALB/c and nude BALB/c, as well as C57Bl/6, gene 2 (Rag2) knockout (KO) C57BL/6, and NOD-SCID mice. The authors observed that in mice lacking T/B responses, 5ASKH developed a larger lesion, with ulceration whereas Friedlin strain induced in these mice smaller lesions, with less inflammation. They also performed experiments employing flow cytometry, and there was a large recruitment of cells to the lesion caused by 5ASKH compared to Friedlin. Concerning cytokines, the results were similar, with an increase in IFN-g, IL-10, TNF and IL-12p40 in the cells from lesions induced by 5ASKH. One intriguing fact is that there was no statistical difference in the parasite load from the lesions caused by the two different strains. Although, the results are interesting, this observation of development of pathology caused by Leishmania has been discussed for a long time, as the author stated, and the results of this manuscript

reinforce this idea.

However, there are some aspects that deserve discussion:

1-There was no explanation about the assay to calculate the parasite load.

**Response**: Parasite burdens in the footpads and draining lymph nodes were quantified by either limiting dilution assay (LDA) or homogenizing tissue in 10 ml of complete medium 199 and incubated in 25cm^2^ culture flasks at 26^o^c for the detection of parasites transformed in vitro from amastigotes to motile promastigotes. At 3 days of culture, promastigotes were counted microscopically. For LDA tissues were homogenized in 1 ml complete medium M199 following 2 fold serial dilution across 96-well culture plates. Culture plates were incubated at 26^o^C. At 1-week culture plates were observed under a microscope for the presence of motile promastigotes. We had included parasite quantification methods in “Materials and Methods” section (now line 153-161) and in figure legends (251-252; 288-290; 495-496; 506-508; 518-520).

2- the amount of cells collected from the footpad of infected mouse. Is this material also used to evaluate the parasite load?

**Response**: No, it was not. The analysis of immune cells from mouse footpad and quantification of footpad parasite burden was done in two separate experiments having similar experimental conditions and infection outcomes.

3- Which type of response was observed in the draining lymph nodes of the mice infected by the two strains of L. major?

**Response**: In wild-type mice, lymphocytes are activated by antigen sensitized APCs in draining lymph node leading to adaptive immune response. It is informative to measure antigen-stimulated re-call response in the draining lymph nodes of the mice infected with *L. major*. In our study we analyzed immune responses in the absence of T/B lymphocytes, where there is no re-call response. We focused on the immune responses induced by *L. major* infection at the site of infection, mice footpad.

4- The authors did not analyze the inflammatory responses before 6 weeks or 4 weeks of infection in some experiments. It would be interesting to perform these experiments, comparing with the wild type mouse.

**Response**: We agree that it is interesting to compare inflammatory responses before 4 or 6 weeks after *L. major* infection. This is one of the limitations of our study (included in the manuscript as a limitation, line 417-420). We will perform a more comprehensive study, in the future, comparing early inflammatory responses with the wild type mouse.

5-One important aspect to discuss, is related to the control of L. major Friedlin in the lesion, since the recruitment of cells is really low and also, the inflammatory cytokines able to activate the macrophages to eliminate the parasite.

**Response**: Thank you for the suggestion. The inflammatory cytokines are able to activate the macrophages for intracellular killing of the parasite. In this study, 5ASKH infection induced higher inflammatory responses compared to Friedlin. However, in the absence of T/B lymphocytes, mice failed to control parasite burden. It indicates that T/B lymphocytes are critical for the control of the parasites, mainly through the production of sufficient amount of critical cytokines, like IFN-γ and TNF-α. It may explain why mice infected with both *L. major* strains showed no difference in parasite burden. However, augmented inflammatory responses after 5ASKH infection may cause the worsen pathology observed (line 393-404).

6- Important data that are not present in the manuscript is related with the source of cytokines in the mice without T/B. Which cell is producing IFN-g?

**Response**: Thank you for the comment. In wild-type mice, T-lymphocytes are the major source of IFN-γ which is central for the protective immunity. In the absence of T/B lymphocytes macrophages, dendritic cells, NK cells, and group 1 innate lymphoid cells may be the source of IFN-γ (Fig 5). However, we are not sure about the source of IFN-γ, which is another limitation of our study (line 394-401).

7- The Discussion needs improvement. It is a summary of results without any discussion. The conclusions are really repetitive.

**Response**: Thank you for the comment. We believe we had improved our discussion and conclusion sections (now line 353, 356-358, 361-369, 373-376, 388-390, 393-407, 409-426, 436-439, 449-462, 465-469, 470, 476-478, 483-484).

Reviewer #3: The study by Abu Musa et al. sought to elucidate how parasite strains influence the role of lymphocytes during Leishmania major infection. To this end, the authors used two strains of differing virulence (Friedlin and 5ASKH) to infect mice that contained or lacked lymphocytes.

Although interesting, I have significant concerns regarding how the authors interpreted their data.

1) Throughout the manuscript, the authors repeatedly claim that while the pathogenesis caused by the Friedlin strain is dependent on lymphocytes, that of the 5ASKH strain is largely not. The authors gone on the argue that the **requirement** for lymphocytes in pathogenesis is strain-dependent. Nonetheless, these statements are not really backed by the data. Instead of portraying a requirement for lymphocytes, the data in Figs 1A and 2A clearly show that lymphocytes influence (either exacerbate or diminish) infection outcome depending on parasite strain. This is further nuanced by the time-dependent development of the lesions, which should be more explicitly described in the results section.

**Response**: We have understood the point well and changed our data interpretation and our current statement is that lymphocytes influence (either exacerbate or diminish) infection outcome depending on *L. major* strains.

2) The finding that lymphocyte presence helps control 5ASKH infection does not mean that pathogenicity is independent of lymphocytes (or the opposite in the case of the Friedlin strain). In order to prove that there really is a requirement for those cells, authors must perform adoptive cell transfer experiments (eg. introduce WT lymphocytes

into nude/RAG2 mice or vice-versa).

**Response**: Thank you for pointing out this precious issue. We appreciate your interpretation and according to your recommendation we have modified our interpretation. Our current statement is lymphocytes influence (either exacerbate or ameliorate) infection outcome depending on *L. major* strains. Based on our current statement, we believe it is not necessary to examine the requirement of T/B lymphocytes for pathogenesis.
